# Cisapride induced hypoglycemia *via* the KCNH6 potassium channel

**DOI:** 10.3389/fendo.2022.1011238

**Published:** 2022-10-17

**Authors:** Jing Lu, Ting-Ting Shi, Sha-Sha Yuan, Rong-Rong Xie, Ru-Xuan Zhao, Juan-Juan Zhu, Jin-Kui Yang

**Affiliations:** ^1^ Beijing Key Laboratory of Diabetes Research and Care, Beijing Diabetes Institute, Beijing Tongren Hospital, Capital Medical University, Beijing, China; ^2^ Department of Endocrinology, Beijing Luhe Hospital, Capital Medical University, Beijing, China

**Keywords:** cisapride, KCNH6, diabetes, insulin secretion, hypoglyacemia

## Abstract

Mutations in *KCNH6* has been proved to cause hypoinsulinemia and diabetes in human and mice. Cisapride is a stomach–intestinal motility drug used to treat gastrointestinal dysfunction. Cisapride has been reported to be a potential inhibitor of the KCNH family, but it remained unclear whether cisapride inhibited KCNH6. Here, we discovered the role of cisapride on glucose metabolism, focusing on the KCNH6 potassium channel protein. Cisapride reduced blood glucose level and increased serum insulin secretion in wild-type (WT) mice fed standard normal chow/a high-fat diet or in db/db mice, especially when combined with tolbutamide. This effect was much stronger after 4 weeks of intraperitoneal injection. Whole-cell patch-clamp showed that cisapride inhibited KCNH6 currents in transfected HEK293 cells in a concentration-dependent manner. Cisapride induced an increased insulin secretion through the disruption of intracellular calcium homeostasis in a rat pancreatic β-cell line, INS-1E. Further experiments revealed that cisapride did not decrease blood glucose or increase serum insulin in KCNH6 β-cell knockout (Kcnh6-β-KO) mice when compared with WT mice. Cisapride also ameliorated glucose-stimulated insulin secretion (GSIS) in response to high glucose in WT but not Kcnh6-β-KO mice. Thus, our data reveal a novel way for the effect of KCNH6 in cisapride-induced hypoglycemia.

## Introduction

Human ether-a-go-go (hERG) belongs to ether-a-go-go family of potassium voltage (Kv)-gated channels (1, 2). Although hERG1 (encoded by KCNH2), 2 (encoded by KCNH6), and 3 (encoded by KCNH7) are sub-members of the hERG potassium channel family, the reliability and gating feature of the voltage of each differ substantially (3). The characteristics of KCNH2 and KCNH7 are similar, but those of KCNH6 are different (3). The KCNH2 gene is mainly expressed in the myocardium, endocrine cells, central nervous system and lymphocytes (3–6). The KCNH6 gene is primarily located in islets and the central nervous system, while the KCNH7 gene is predominantly expressed in dorsal root ganglia and islets (7–11). There are fewer functional studies of KCNH6 and KCNH7 than KCNH2. One previous report revealed that KCNH2 might activate cardiac delayed rectifier current (4). Type 2 diabetes mellitus (T2DM) patients who have mutations of KCNH2 caused long QT syndrome (LQTS) and increasing insulin secretion (12).

ATP-sensitive potassium channel (K_ATP_) depolarization channel and other potassium repolarization channels regulate insulin secretion by pancreatic β-cells (13). Some drugs, such as tolbutamide, a sulfonylurea, induce hypoglycemia by blocking K_ATP_ channels in pancreatic β-cells (14). Another study revealed that the KCNH repolarization channel regulated insulin secretion and the firing of human β cells (15). Previously, our group found that adult patients with hypoinsulinism and diabetes presented with a heterozygous mutation in the KCNH6 gene (16). KCNH subtype expression in different tissue types can distinguish members of this gene family at the genetic level; however, specific inhibitors for each gene are lacking. Inhibitors such as E4031 and dofetilide can only be used to inhibit the hERG family as a whole and are not specific to individual subtypes (17, 18). Our group found that berberine, a Kcnh6 inhibitor, promoted high glucose-dependent insulin secretion (19).

Cisapride is a stomach–intestinal motility drug widely used to treat gastrointestinal dysfunction. Previous reports revealed that cisapride overdose can cause LQTS and fatal arrhythmia. Thus, Cisapride was voluntarily removed from the U.S. market and is limited in Europe. KCNH2 participates in the repolarization of myocardial action potentials. Thus, cisapride is a potential inhibitor of the KCNH family, especially KCNH2. However, whether cisapride also inhibits KCNH6 remains unclear.

Here, we evaluated the effect of the gastrointestinal prokinetic agent cisapride on KCNH6. Animal experiments demonstrated that cisapride can down-regulate blood glucose levels and up-regulate insulin secretion, especially when combined with tolbutamide. Whole-cell patch-clamp showed that cisapride inhibited KCNH6 currents in transfected HEK293 cells. Further experiments revealed that cisapride did not decrease blood glucose or increase serum insulin in KCNH6 β-cell knockout (Kcnh6-β-KO) mice. These results identify new insights into the therapeutic value of KCNH6-targeted drugs.

## Method

### Animals

Male C57BL/6J and db/db mice purchased from Jiangsu Gempharmatech Company (Jiangsu, China) were used in the experiments. Kcnh6 β-cell conditional knockout (Kcnh6-β-KO) mice were constructed as before (19). Wild-type (WT) and Kcnh6-β-KO mice used in this study were eight weeks old. The mice were kept in indiviual plastic cages with a 12 h light/dark cycle at room temperature.

Animal experiments were approved by of the Ethical Review Committee at Capital Medical University on laboratory Animal Care (No. TRECKY2018-037).

### Reagents

Tolbutamide and cisapride were both bought from Sigma–Aldrich (Saint Louis, USA). E4031 and dofetilide were purchased from MedChemExpress.

### Cells

INS-1E cells purchased from Cell Resource Center (Beijing, China) were kept in MEM culture (HyClone) with 10% fetal bovine serum (FBS).

Islets from mice were isolated as previously described and grown in RPMI 1640 medium for 24 hours with 11 mmol/L glucose (Gibco, USA) 10% FBS, 1% penicillin/streptomycin at 37°C in a 5% CO_2_ atmosphere (20). Over 100 islet cells were seeded in 0.05% trypsin‐0.02% EDTA solution at 37°C for 10 min and completely dispersed into single pancreatic islet β-cells. An insulin ELISA kit (Millipore, MA, USA) was used to measure insulin.

All the cells were maintained at 37°C in an incubator with 5% CO_2_ within 30 hours of plating.

### Plasmids

Human *KCNH6* gene was cloned into a pcDNA3.1 vector (Sino-GenoMax, Shanghai, China). Lipofectamine 3000 (Invitrogen, USA) was used to transfect HEK293 cells with plasmid DNA following the manufacturer’s protocol. 293T cells were transfected with different plasmids for 48 hours before the next experiment.

### Metabolic experiment

For this procedure, intraperitoneal glucose tolerance tests (IPGTTs) and intraperitoneal insulin release tests (IPIRTs) were used. Detailed methods were as previously described (20). The mice were fasted except for water for 8 h before the tests were conducted. The mice were administered the different drugs intragastrically before being given 50% glucose at the dosage of 2 g/kg of body weight (i.p.). Glucose taken from the tail vein was measured before injection (time 0) and 15, 30, 60, and 120 minutes after injection with a handheld glucometer (One-Touch Ultra, Johnson, USA). An ELISA kit (Millipore, MA, USA) was used to measure insulin.

Intraperitoneal insulin tolerance tests (IPITTs) were used on 4-hour-fasted mice after 12 weeks on a HFD diet. Insulin was injected (1 U/kg, i.p.) before blood glucose was detected at 15, 30, 60, and 120 minutes. The area under the curve (AUC) of the IPGTT and IPITT was considered and compared between different groups.

### Cytosolic Ca^2+^ measurements

The intracellular Ca^2+^ concentration in INS-1E cells was determined with a confocal laser scanning microscope and 5 μM calcium orange (MA, USA, Invitrogen). Calcium orange was dissolved in Hanks buffer with additional Pluronic F-127 (0.005%, Molecular Probes, MA, USA) at 37°C for 30 min and subsequently washed twice using Hanks buffer. Fluorescence measurements were carried out using anargon ion laser (excitation: 561 nm). Images were recorded every 2 s using a DeltaVision Ultra system (GE, Massachusetts, USA). Baseline fluorescence (F0) was determined by averaging 20 images. Then cells were transferred in the KRBB buffer with 16.7mmol/l glucose, images were recorded every 2 s for 15min. Fractional fluorescence (F/F0) indicates the changes of intracellular calcium concentration. After the base line were stable for 5 min, the intracellular Ca^2+^ concentration was measured for another 5 min with Control (Ctrl), 10 mM tolbutamide (T), cisapride (C) or tolbutamide+cisapride (T+C) under the stimulation with different glucose stimulation. PBS was used as a control. AUC was measured before (0-5 min) and after (5-10 min) different drugs application. Statistical comparisons were calculated using the Wilcoxon matched pairs test (n=4).

### Glucose-stimulated insulin secretion

10 size-matched islets were incubated with 2.8 mM glucose Krebs–Ringer buffer (KRB) adjusted to pH 7.3 with HEPES for 60 min at 37°C. After incubation in low-glucose KRB for 60 min, the islets were stimulated in culture medium containing 2.8 mM or 16.7 mM glucose with different concentrations of inhibitors or with the vehicle for an additional 60 min at 37°C. An insulin ELISA detection kit from Mercodia (Uppsala, Sweden) was used to measure insulin.

### Electrophysiology

To evaluate the effects of cisapride on hERG current amplitudes, whole-cell patch-clamp experiments were performed as previously described (16). Whole-cell configuration involved use of a glass pipette with tip resistance of 2.5–4.0 MΩ filled with the internal pipette solution. The mean series resistance was not more than 15 MΩ. Data were collected at least 10 min after incubating samples with compounds or the vehicle to record the currents of HEK293 cells transiently expressing human KCNH6. The pipetted solution contained (in mmol/L) KCl 20, K-aspartic 115, HEPES 10, MgCl_2_ 1, EGTA 5, and Na_2_-ATP 2 (adjusted to pH 7.3 with KOH). The records were gathered *via* an EPC-10 amplifier and stored in PatchMaster software (HEKA, Germany).

A glass pipette was pulled using a micropipette puller. A micromanipulation device was used to manipulate the glass pipette under a microscope. After touching the glass pipette to the cell membrane, a strong seal of 1 GΩ was achieved by slight suction. After this strong seal was established, we calculated the fast capacitance (in pF) compensation, and the membrane was disrupted. After achieving the whole-cell mode, we used whole-cell capacitance compensation to compensate for the cell capacitance.

The cells were cultured with the test compound until a stable current was reached. The test and control solutions were then fluxed into the chamber *via* a gravity-fed delivery system. The current detected by each cell in the external solution without compound was used as that cell’s blank control. The external solution without the test compound was used as a blank control. All experiments were performed at room temperature.

Kv currents were measured at a stable potential of -80 mV and then recorded while the current was increased from -70 mV to +70 mV in increments of 10 mV. The mean membrane capacitance was 18.53 ± 2.09 pF (n = 5) and the mean access resistance was 11.8 ± 1.89 MΩ (n = 5). Representative of three independent experiments were shown in the figure.

### Statistical analysis

Data are presented as the mean ± standard error of the mean (SEM). Statistical comparisons were calculated using Mann–Whitney *U* test or Wilcoxon matched pairs test for experiments (GraphPad Prism, version 8.0). *P* value < 0.05 was considered to represent significance.

## Results

### Cisapride combined with tolbutamide protect glucose metabolic in wild-type mice fed normal chow

**Figure 1:**
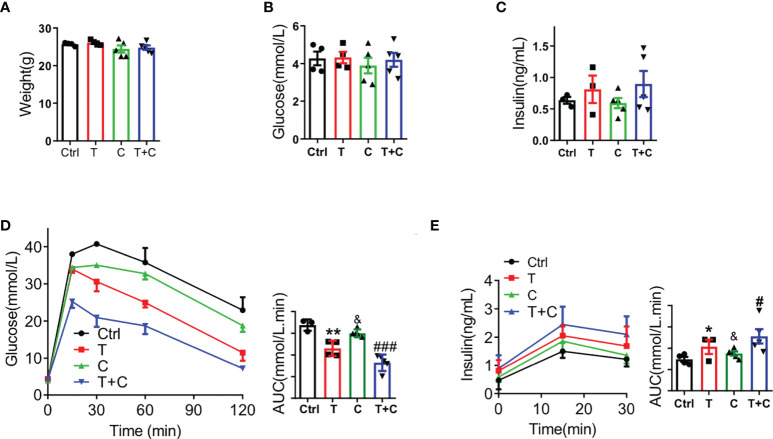
Cisapride combined with tolbutamide regulated glucose metabolism in wild-type mice Wild-type (WT) mice were divided based on **(A)** body weight, **(B)** fasting blood glucose and **(C)** fasting serum insulin. **(D)** Different drugs were administered transiently to WT mice, including the control (physiological saline, Ctrl), tolbutamide (T, 20 mg/kg), cisapride (C, 20 mg/kg) and tolbutamide combined with cisapride (T+C). Intraperitoneal glucose tolerance tests (IPGTTs) were performed in the 4 groups. **(E)** Intraperitoneal insulin release tests (IPIRT) were performed. n=6 for each group **(A–E)** ^&^ *P* < 0.05, ^**^ *P* < 0.005, *^#^P < 0.05*, ^###^ *P* < 0.005 vs. Ctrl. Statistical comparisons were calculated using the Mann–Whitney *U* test **(A–E)**.

To demonstrate the role of cisapride in metabolism, we used male wild-type (WT) mice fed normal chow for 12 weeks. The body weight was calculated first to ensure that there was no significant difference at the initiation of the study (**Figure 1A**). Fasting blood glucose and serum insulin were also measured and showed no difference (**Figures 1B, C**). The WT mice were then randomly divided into control (Ctrl), tolbutamide (T), cisapride (C) and tolbutamide combined with cisapride (T+C) groups. Physiological saline was used as a negative control. In the IPGTT and IPIRT, tolbutamide (20 mg/kg) reduced blood glucose and increased serum insulin compared with the control group. Cisapride (10 mg/kg) showed the same tendency, but the differences were not significant. The area under the concentrations of both blood glucose and insulin secretion differed the most in the group treated with tolbutamide combined with cisapride (**Figures 1D, E**). Our above-mentioned data demonstrate that cisapride alone had a weak effect on insulin secretion but significantly increased insulin secretion when combined with tolbutamide.

### Cisapride alleviated glucose metabolism in wild-type mice fed a high-fat diet

**Figure 2:**
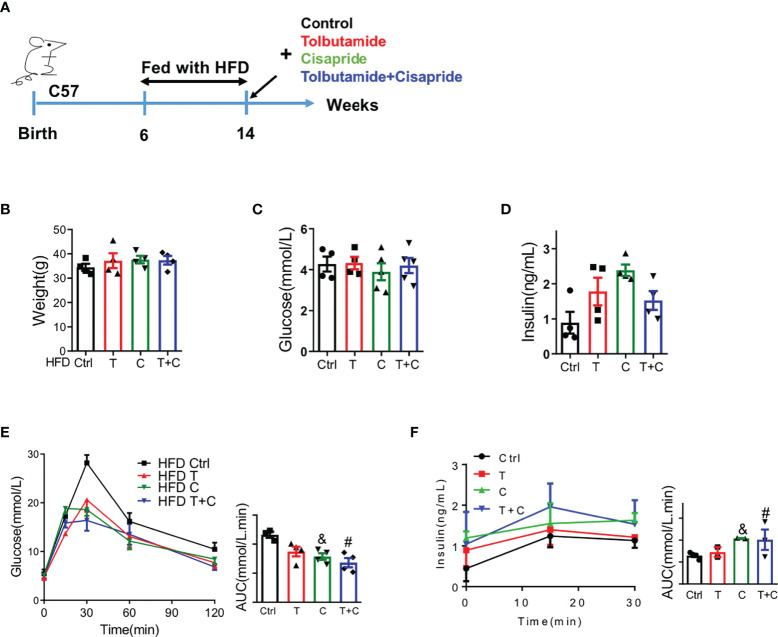
Cisapride regulated glucose metabolism in wild-type mice fed a high-fat diet **(A)** Wild-type (WT) mice were fed a high-fat diet (HFD) for 8 weeks before the experiment. **(B)** Body weight, **(C)** fasting blood glucose and **(D)** fasting serum insulin were measured. **(E)** Different drugs were administered transiently to WT mice, including the control (physiological saline, Ctrl), tolbutamide (T, 20 mg/kg), cisapride (C, 10 mg/kg) and tolbutamide combined with cisapride (T+C). Intraperitoneal glucose tolerance tests (IPGTTs) were performed in the 4 groups. **(F)** Intraperitoneal insulin release tests (IPIRT) were performed. n=5 for each group **(B–F)** ^&^P < 0.05, ^#^ *P* < 0.05 vs. Ctrl. Statistical comparisons were calculated using the Mann–Whitney *U* test **(A–E)**.

Next, we sought to determine the function of cisapride using WT mice fed a high-fat diet (HFD) in transient and long-term experiments. Male WT mice were fed with HFD for 8 weeks before the IPGTT and IPIRT experiments (**Figure 2A**). Body weight, fasting blood glucose and fasting serum insulin were detected first to confirm that there were no initial differences among the four groups (**Figures 2B–D**). The cisapride group of WT mice fed a HFD had decreased blood glucose and increased insulin. When combined with tolbutamide, the phenomenon was aggravated (**Figures 2E, F**).

**Figure 3:**
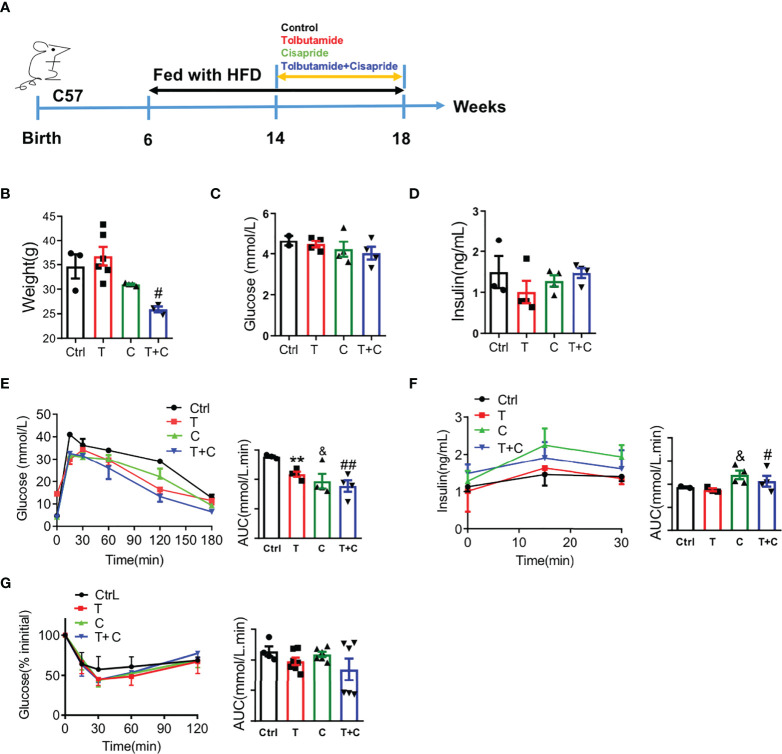
Long-term administration of cisapride regulated glucose metabolism in wild-type mice fed a high-fat diet **(A)** Wild-type (WT) mice were fed a high-fat diet (HFD) for 8 weeks before a 4-week daily administration of different drugs. Mice were divided into four groups: control (physiological saline, Ctrl), tolbutamide (T), cisapride **(C)** and tolbutamide combined with cisapride (T+C). **(B)** Body weight, **(C)** fasting blood glucose and **(D)** fasting serum insulin were measured. **(E)** Intraperitoneal glucose tolerance tests (IPGTTs) were performed. **(F)** Intraperitoneal insulin release tests (IPIRT) were performed. **(G)** Intraperitoneal insulin tolerance tests (IPITTs) were performed. n=5 for each group **(B–G)** ^&^P < 0.05, ^#^P < 0.05, ^##^ *P* < 0.005 vs. Ctrl, ^###^ *P* < 0.005 vs. Ctrl. Statistical comparisons were calculated using the Mann–Whitney *U* test **(A–F)**.

For the long-term experiment, cisapride, tolbutamide, tolbutamide combined with cisapride and the control were administered intragastrically daily for 4 weeks (**Figure 3A**). Body weight was reduced in the tolbutamide combined with cisapride group (**Figure 3B**). Fasting glucose and insulin were similar among the four groups (**Figures 3C, D**). Unlike in the transient experiment, cisapride-treated mice showed better glucose tolerance and higher insulin secretion than tolbutamide-treated animals (**Figures 2E, F**). IPITT was performed to measure insulin tolerance in the four groups, and no significant differences were observed (**Figure 2G**). Thus, our experiment shows that long-term administration of cisapride can significantly reduce glucose concentrations and increase insulin secretion in WT mice fed a HFD.

### Cisapride ameliorated glucose metabolism in db/db mice

**Figure 4:**
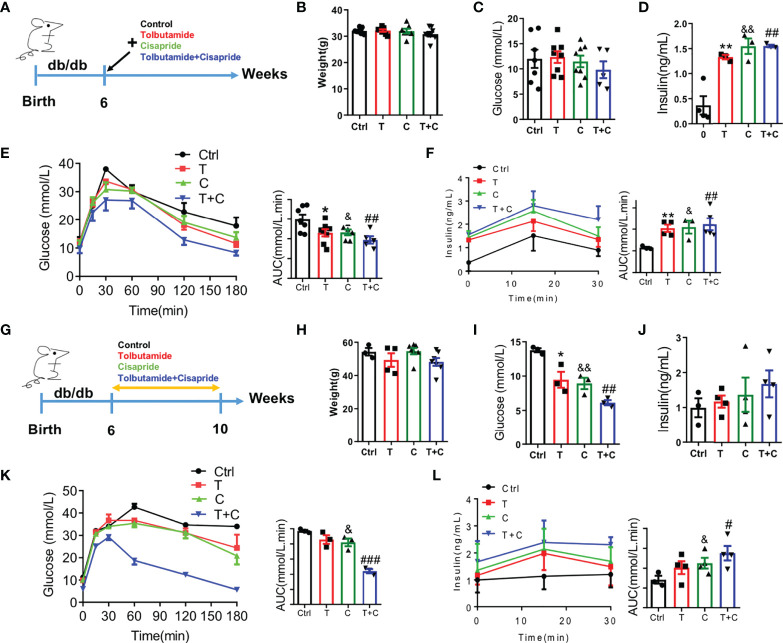
Cisapride regulated glucose metabolism in db/db mice **(A)** Six-week-old db/db mice were divided based on **(B)** body weight, **(C)** food intake, **(D)** fasting blood glucose and **(E)** fasting serum insulin. Different drugs were administered transiently to WT mice, including the control (physiolgical saline, Ctrl), tolbutamide (T, 20 mg/kg), cisapride (C, 20 mg/kg) and tolbutamide combined with cisapride (T+C). **(F)** Intraperitoneal glucose tolerance tests (IPGTTs) were performed in the 4 groups. **(G)** The abovementioned drugs were administered daily for 4 weeks to six-week-old db/db mice. **(H)** body weight, **(I)** food intake, **(J)** fasting blood glucose and **(E)** fasting serum insulin. **(K)** IPGTTs were performed. **(L)** IPIRT was performed. n=4 **(B–F)** or n=3 **(G–L)** for each group. ^*^ *P* < 0.05, ^**^ *P* < 0.01, ^&^ *P* < 0.05, ^&&^ *P* < 0.01, ^#^ *P* < 0.05, ^##^P < 0.01, ^###^P < 0.005 vs. Ctrl. Statistical comparisons were calculated using the Mann–Whitney *U* test **(B-F, G–L)**.

As is well-known that db/db mice have been used as type 2 diabetes models, we chose db/db mice here as a positive control. Male db/db mice at the age of 6 weeks were used in the experiment (**Figure 4A**). Body weight and fasting blood glucose were detected to confirm that there were no initial differences among the four groups (**Figures 4B, C**). Fasting insulin secretion were different between control and the three other groups (**Figure 4D**). The cisapride and tolbutamide groups demonstrated significantly reduced blood glucose and increased insulin secretion levels. When cisapride was combined with tolbutamide, blood glucose was reduced, and insulin secretion was increased (**Figures 4E, F**).

Next, cisapride, tolbutamide, and cisapride combined with tolbutamide were administered for 4 weeks to db/db mice (**Figure 4G**). Fasting blood glucose and fasting serum insulin levels represent the sensitivity to insulin (21). The db/db mice showed lower fasting blood glucose levels after 4 weeks of drug administration than the control group (**Figures 4H–J**). Cisapride administration significantly reduced blood glucose and increased serum insulin, suggesting that cisapride could reduce hyperglycemia in db/db mice (**Figures 4K, L**). The results underly the protective effect of cisapride on glucose metabolism in db/db mice.

### Cisapride inhibited the hERG current in transfected HEK293 cells

**Figure 5:**
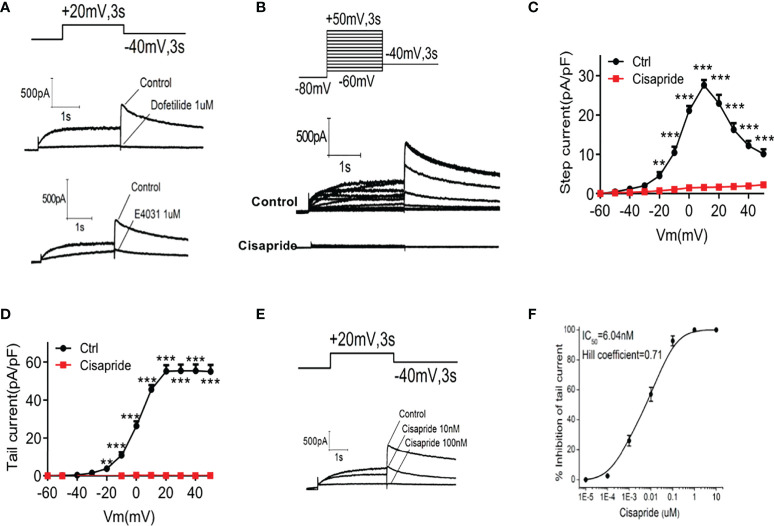
Cisapride inhibited HERG channels in transfected HEK293 cells HERG currents were recorded in transfected HEK293 cells. Cells were voltage-clamped at −80 mV for 1 s, depolarized from −60 mV to +50 mV for 3 s and repolarized to −40 mV for 3 s under stimulation with **(A)** E4031, dofetilide or **(B)** cisapride. **(C)** The time-dependent step current and **(D)** peak tail current I-V curves were recorded before and after cell perfusion with 1 𝜇mol/L cisapride. **(E)** Cells were depolarized to a voltage of +20 mV for 3 s using a stepped procedure. Peak tail currents were recorded at different cisapride concentrations (10 and 100 nmol/L). **(F)** The IC50 of cisapride was calculated. The concentration−response curve was fitted to a Hill equation. n=4 for each group **(A–F)**. ^**^ *P* < 0.01, ^***^ *P* < 0.005 vs. Ctrl.

We conducted electrophysiological experiments to determine whether cisapride can directly inhibit the hERG current in HEK293 cells. The transfection rate was nearly 90% according to the Western of Kcnh6 (**Supplementary Figure 1A**). Cells transfected with hERG were maintained at -80 mV for 1 s before depolarization for 3 s. Tail current was induced at −40 mV for 3 second at least four cells. Peak tail current was recorded at 10 mV intervals from -60 mV to +50 mV, which meant that the HERG channel was open at that voltage. E4031 and dofetilide were used as inhibitor controls (**Figure 5A**). After perfusion with 1 𝜇mol/L cisapride for 5 min, the current was elicited (**Figure 5B**). The data curves showed that 1 𝜇mol/L cisapride reduced the pA/pF of peak tail currents significantly at a time-dependent manner. When the potential was at 50mv, in control condition, the tail current density was 55.36 ± 7.4 pA/pF (n = 5) and could be suppressed in the presence of 1 µmol/l cisapride to 0.11 ± 0.04 pA/pF (n = 5; ^***^ *p <*0.001, unpaired-tested) (**Figures 5C, D**). Tail currents decreased when the concentrations of cisapride increased, indicating that inhibition of cisapride was concentration dependent. HERG channel currents were completely inhibited by 100 nmol/L cisapride (**Figure 5E** and **Supplementary Figures 2A–D**). A dose−response curve was constructed, and the results were fitted to a Hill equation. The IC50 of cisapride was 6.04 nM. The Hill slope, n_H_, was calculated as 0.71 (**Figure 5F**).

### Cisapride combind with tolbutamide improved intracellular Ca^2+^ concentrations under high glucose conditions

**Figure 6:**
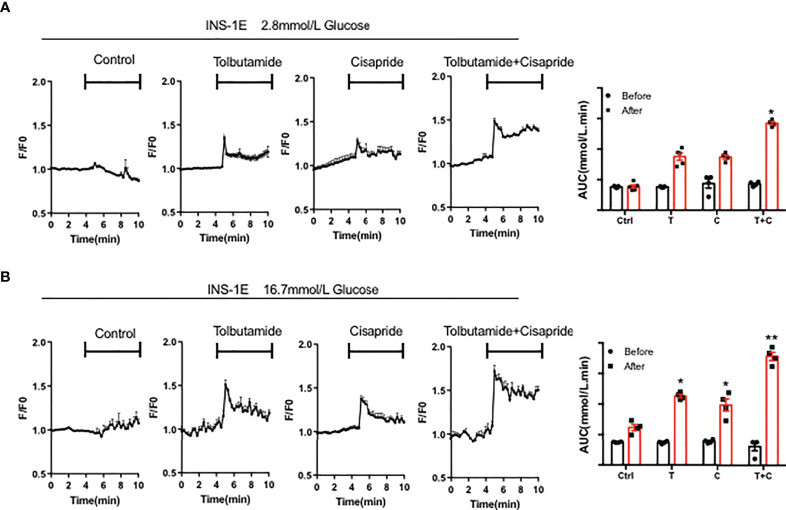
Cisapride affected the intracellular Ca^2+^ concentration in INS-1E cells The intracellular Ca^2+^ concentration was measured in the rat pancreatic cell line INS-1E treated with Control (Ctrl), 10 mM tolbutamide (T), cisapride **(C)** and tolbutamide+cisapride (T+C) under the stimulation with **(A)** 2.8 mM glucose and **(B)** 16.7 mM glucose for 5 min before the base line was stable for 5 min. PBS was used as a control. AUC was measured before (0-5 min) and after (5-10 min) different drugs application. n=4 **(A-B)** for each group. ^*^ *P* < 0.05, ^**^ *P* < 0.005 vs. Ctrl. Statistical comparisons were calculated using the Wilcoxon matched paired-test **(A, B)**.

The Ca^2+^ concentration triggers insulin secretion in islet β-cells directly. The glucose-mediated increase in Ca^2+^ in islets was also measured in a rat pancreatic cell line, INS-1E. PBS was used as a negative control. 10mM tobutamide, cisapride and tolbutamide combined with cisapride were used after the base line was stable. AUC was measured before and after different drugs application. The results showed that neither tolbutamide nor cisapride can significantly increase the Ca^2+^ concentration at 2.8 mM glucose (**Figure 6A**). Further experiment demonstrated that when combined with tolbutamide, cisapride showed the highest increasing intracellular Ca^2+^ concentrations at 16.7mM glucose (n = 4; ^**^ *p <*0.005, Wilcoxon matched paired-test) (**Figure 6B**). Thus, we concluded that when cisapride combined with tolbutamide can improve intracellular Ca^2+^ concentrations under high glucose conditions.

### Cisapride increased insulin secretion in WT but not Kcnh6-β-KO mice

**Figure 7:**
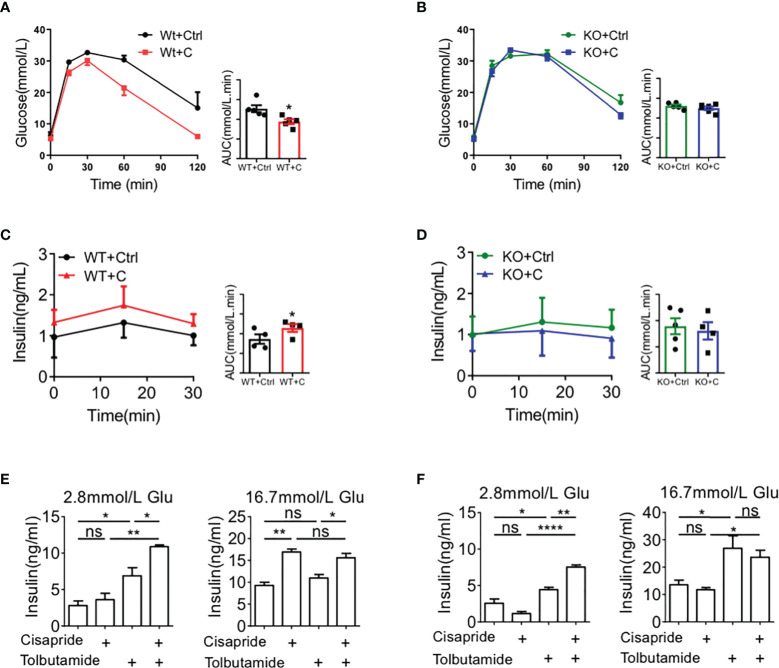
Effects of cisapride on WT and Kcnh6-β-KO mice *in vivo* and *in vitro* **(A, B)** Blood glucose and plasma insulin levels in WT and cisapride-treated WT mice. **(C, D)** Blood glucose and plasma insulin levels in Kcnh6-β-KO and cisapride-treated Kcnh6-β-KO mice. **(E)** Islets from WT and **(F)** Kcnh6-β-KO mice were treated with 10 mM cisapride or the vehicle (control) while being stimulated with 25 mM glucose for 30 min. The secreted insulin level in the supernatant was normalized to the total insulin content in the islets. n=4 for each group. ^*^ *P* < 0.05, ^**^ *P* < 0.005 vs. Ctrl. Statistical comparisons were calculated using the Mann–Whitney *U* test **(A–F)**. NS, Non Significance.

Next, we investigated the effects of cisapride in two pairs, Kcnh6-β-KO mice + WT mice, by conducting an IPGTT and IPIRT. The IPGTT and IPIRT revealed that when cisapride was administered, blood glucose was decreased and serum insulin was increased in WT mice fed standard glucose under glucose loading (**Figures 7A, B**). However, blood glucose and serum insulin secretion were almost the same in Kcnh6-β-KO mice administered cisapride (**Figures 7C, D**). Tolbutamide was also administered to WT and Kcnh6-β-KO mice. Tolbutamide reduced blood glucose in both WT and Kcnh6-β-KO mice, demonstrating that tolbutamide did not affect KCNH6 protein (**Supplementary Figures 3A, B**). Thus, we concluded that cisapride stimulated the increase in insulin secretion in WT but not Kcnh6-KO or Kcnh6-β-KO mice.

Furthermore, GSIS was assessed using the islets of WT and Kcnh6-β-KO mice to measure the function of cisapride on Kcnh6. Cisapride increased the GSIS of islets from WT mice at different levels (**Figures 7E, F**). When combined with tolbutamide, cisapride increased insulin secretion more significantly. However, the drug did not affect islets from Kcnh6-β-KO mice (**Figures 7G, H**).

## Discussion

We evaluated the role of cisapride on the KCNH6 channel current. The glucose tolerance test demonstrated that cisapride reduced blood glucose and increased serum insulin in WT mice fed a normal chow/high-fat diet as well as in db/db mice, especially when combined with tolbutamide. The effect was much stronger after intragastric administration for 3 months. Cisapride administration resolves increasing insulin secretion by disruption of intracellular calcium homeostasis. Whole-cell patch-clamp showed that cisapride inhibited KCNH6 currents in transfected HEK293 cells with a concentration-dependent manner. The results revealed that cisapride improves GSIS upon high glucose (16.7 mmol/L) vs. low glucose (2.8 mmol/L). Meanwhile, cisapride did not decrease blood glucose in Kcnh6 β-cell conditional knockout mice compared to wild-type mice or *in vitro*. Thus, KCNH6 plays a cruial role in cisapride-induced hypoglycemia.

Glucose metabolism can cause an initial increase in the ATP/ADP ratio, then the K_ATP_ channel closed and also the plasma membrane depolarized. Then, the voltage-gated calcium channel (VDCC) opens, and different ions flow into the cell and trigger insulin secretion. The repolarization stage of the action potential is vital for insulin secretion. Outward potassium currents drive the repolarizing stage and modulate insulin release (13). Although various depolarization K^+^ channels have been detected in pancreatic β cells, their individual contributions to insulin secretion remain unknown (22). Here, we administered tolbutamide combined with cisapride in animal experiments. Tolbutamide is a well-known inhibitor of β-cell K_ATP_ channels that causes plasma membrane depolarization to trigger the influx of Ca^2+^ and subsequent insulin release. Cisapride can inhibit the hERG depolarization K^+^ channel (23). The insulin response was markedly higher in all animal models treated with tolbutamide combined with cisapride. This observation suggests that hERG appears to be required in insulin secretion.

Previously, our group found that dysfunction of the *KCNH6* gene caused hypoinsulinemia and hyperglycemia. First, an increasing intracellular calcium level was observed in mice and adult patients. Sustained elevations in cytoplasmic Ca^2+^ levels may activate programmed cell death. Intracellular Ca^2+^ overload is a widely driver of cell death in different tissues including neurons, cardiomyocytes, and pancreatic β cells. Here, we found that cytosolic Ca^2+^ levels were increased in INS-1E cell lines with cisapride administration, especially when combined with tolbutamide, under high glucose conditions (16.7 mM), suggesting that cisapride may increase insulin secretion *via* intracellular calcium stimulation.

We next used the whole-cell patch-clamp technique to explore the function of cisapride on KCNH6 channel currents. The path clamp experiment is the “gold standard” for examining different channel functions in different cells (24, 25). The KCNH inhibitors E-4031 and dofetilide were used as controls to assess the impact on channel to be reduced or completely inhibited. We found that the tail currents of KCNH6 decreased upon treatment with 1𝜇mol/L cisapride, indicating that cisapride effectively prevent the KCNH6 channel in transfected HEK293 cells. When the concentration of cisapride increased, the tail currents of KCHN6 were reduced and completely inhibited at 100𝜇mol/L cisapride. Thus, we conclude that cisapride inhibition was concentration dependent.

In this study, we used mouse model instead of human according to the drug effect on the function of hERG channels. Here, 20 mg/kg cisapride may reduce blood glucose levels in WT mice with HFD according to the IPGTT experiment. An increase in insulin secretion was also detected in IPIRT experiment. However, cisapride did not change blood glucose in Kcnh6-β-KO mice. We also performed an animal experiment using tolbutamide in Kcnh6-β-KO mice. Tolbutamide reduced glucose and increased insulin in both WT and Kcnh6-β-KO mice. These above-mentioned results suggest that cisapride can reduce blood glucose and increase serum insulin targeting the KCNH6 protein.

Although our study revealed that KCNH6 plays an important role in cisapride-induced hypoglycemia, there are still some limitations. Some of the Food and Drug Administration (FDA)-approved drugs, such as grepafloxacin, cisapride and terodiline, have been withdrawn from the major market in some countries because of their effect on the function of hERG channels (26–28). A case report concluded that even as monotherapy, cisapride may pose dangers for high-risk diabetic patients (29). Although cisapride may even not usable in gastric emptying in diabetic gastroparetic dogs, it is available in the United States and Canada for use in animals (30). Studies on reducing the side effects while maintaining the efficiency of binding to targets may allow some drugs to return to the market. Here, we chose cisapride as a model hERG blocker because of its potential to be improved and remarketed. However, the target structure must be optimized to meet its possible future directions regarding veterinary uses of cisapride (31, 32). Second, cisapride was reported to be an antagonist of 5-hydroxytryptamine (5-HT)_4_ receptor (5-HT_4_R) (33). We should exclude this effect on pancreatic β-cells in future work.

In summary, we demonstrated that cisapride combined with tolbutamide might reduce blood glucose and serum insulin levels in different animal models, especially in WT mice fed a HFD or db/db mice, after long-term administration. Further experiments showed that KCNH6 played a key role in cisapride-induced hypoglycemia. Our study may provide new insights into the therapeutic value of KCNH6-targeted drugs.

## Data availability statement

The original contributions presented in the study are included in the article/**Supplementary Material**. Further inquiries can be directed to the corresponding author.

## Ethics statement

The animal study was reviewed and approved by Ethical Review Committee at Capital Medical University on laboratory Animal Care.

## Author contributions

J-KY and JL conceived and designed the study. JL, T-TS and S-SY designed and performed the experiments and wrote the draft of the manuscript. J-KY is the guarantor of this work. All authors contributed to the article and approved the submitted version.

## Funding

This work was supported by grants from the National Natural Science Foundation of China (81930019) to J-KY, and the National Natural Science Foundation of China (81800688, 82070890) to JL, and Beijing Municipal Administration of Hospitals Incubating Program (PX2019006) to JL.

## Acknowledgments

The authors thank the participants and staff of the studies for valuable contributions.

## Conflict of interest

The authors declare that the research was conducted in the absence of any commercial or financial relationships that could be construed as a potential conflict of interest.

## Publisher’s note

All claims expressed in this article are solely those of the authors and do not necessarily represent those of their affiliated organizations, or those of the publisher, the editors and the reviewers. Any product that may be evaluated in this article, or claim that may be made by its manufacturer, is not guaranteed or endorsed by the publisher.

## Supplementary material

Supplementary Figure 1 Kcnh6 expression of transfected HEK293 cellsWestern blot was done to show the rate of transfection. ^***^ *P* < 0.001 vs. Ctrl. n=3 for each group.

Supplementary Figure 2 Different concentrations of cisapride inhibited HERG channels in transfected HEK293 cells Representative figures of different concentrations, including **(A)** 10 nM, **(B)** 100 nM, **(C)** 1𝜇mol/L and **(D)** 10𝜇mol/L cisaprid, that induced hERG currents in transfected HEK 293 cells. n=4 for each group.

Supplementary Figure 3
**(A)** Blood glucose in WT and tolbutamide-treated WT mice. **(B)** Blood glucose in Kcnh6-β-KO and tolbutamide-treated Kcnh6-β-KO mice. n=5 for each group. *P < 0.05, **P < 0.005 vs. Ctrl. Statistical comparisons were calculated using the Mann–Whitney U test **(A, B)**.
