# Supplementary figures and images for: Cisapride induced hypoglycemia via the KCNH6 potassium channel
Source: Front Endocrinol (Lausanne). 2022 Oct 17;13:1011238. doi: 10.3389/fendo.2022.1011238 (PMC9618959; doi:10.3389/fendo.2022.1011238)

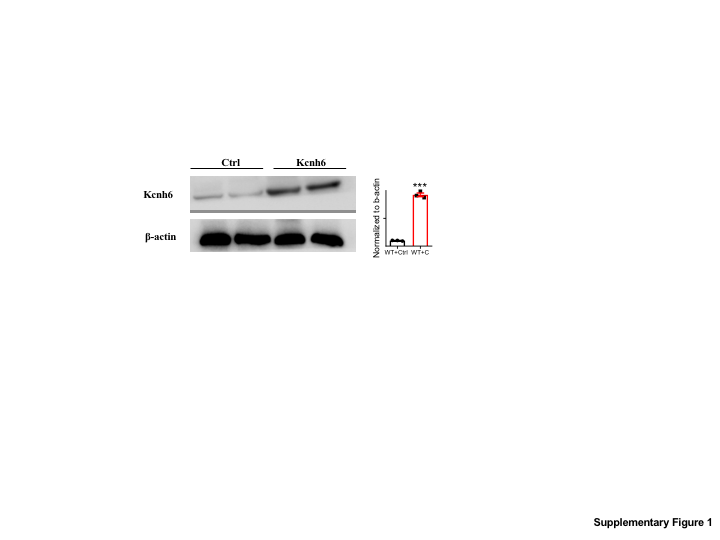

Supplement: Supplementary Figure 1 — Kcnh6 expression of transfected HEK293 cellsWestern blot was done to show the rate of transfection. *** P < 0.001 vs. Ctrl. n=3 for each group. [file Image_1.tiff]

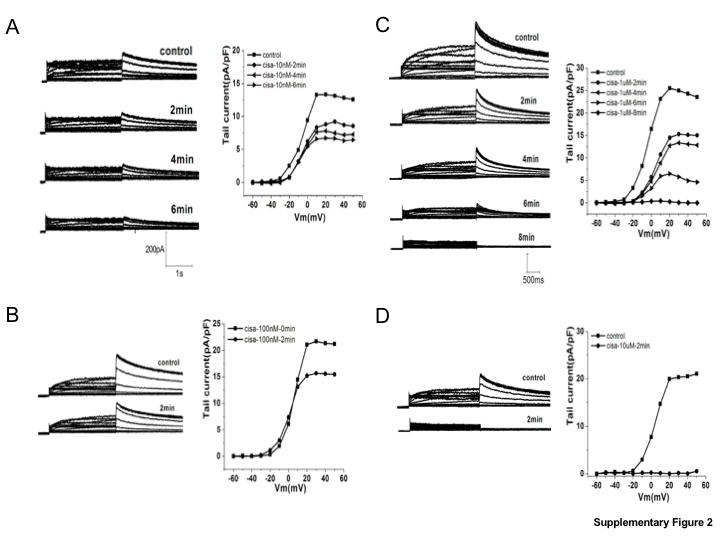

Supplement: Supplementary Figure 2 — Different concentrations of cisapride inhibited HERG channels in transfected HEK293 cells Representative figures of different concentrations, including (A) 10 nM, (B) 100 nM, (C) 1𝜇mol/L and (D) 10𝜇mol/L cisaprid, that induced hERG currents in transfected HEK 293 cells. n=4 for each group. [file Image_2.tiff]

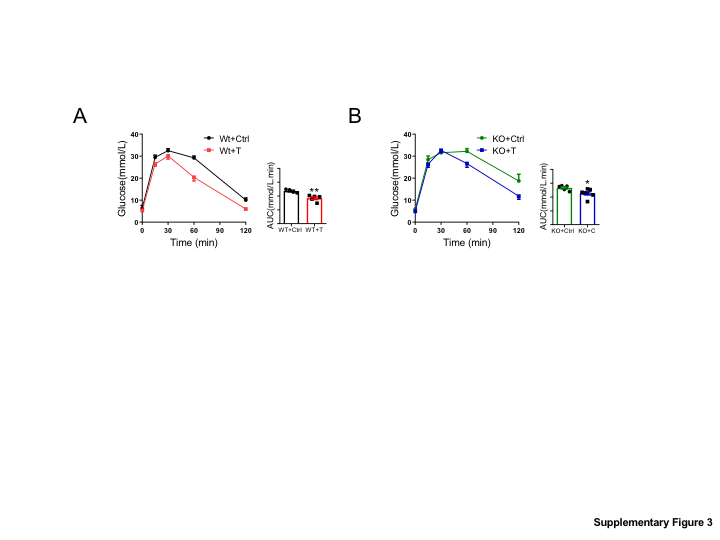

Supplement: Supplementary Figure 3 — (A) Blood glucose in WT and tolbutamide-treated WT mice. (B) Blood glucose in Kcnh6-β-KO and tolbutamide-treated Kcnh6-β-KO mice. n=5 for each group. *P < 0.05, **P < 0.005 vs. Ctrl. Statistical comparisons were calculated using the Mann–Whitney U test (A, B). [file Image_3.tiff]
